# “My People Already Know That”: The Imagined Audience and COVID-19 Health Information Sharing Practices on Social Media

**DOI:** 10.1177/20563051221122463

**Published:** 2022-09-20

**Authors:** Jaigris Hodson, Victoria O’Meara, Christiani Thompson, Shandell Houlden, Chandell Gosse, George Veletsianos

**Affiliations:** 1 Royal Roads University, Canada; 2 University of Western Ontario, Canada; 3 University of Saskatchewan, Canada

**Keywords:** Keywords, Imagined audience, misinformation, health science communication, social media sharing practices

## Abstract

This article examines how imagined audiences and impression management strategies shape COVID-19 health information sharing practices on social media and considers the implications of this for combatting the spread of misinformation online. In an interview study with 27 Canadian adults, participants were shown two infographics about masks and vaccines produced by the World Health Organization (WHO) and asked whether or not they would share these on social media. We find that interviewees’ willingness to share the WHO infographics is negotiated against their mental perception of the online audience, which is conceptualized in three distinct ways. First, interviewees who would not share the infographics frequently describe a self-similar audience of peers that are “in the know” about COVID-19; second, those who might share the infographics conjure a specific and contextual audience who “needs” the information; and finally, those who said they would share the infographics most frequently conjure an abstract audience of “the public” or “my community” to explain that decision. Implications of these sharing behaviors for combatting the spread of misinformation are discussed.

## Introduction: COVID-19 Information Sharing on Social Media

According to reports from BuzzFeed News, in the 3 months leading up to the 2016 US presidential election, the top 20 “fake news” stories on Facebook were shared more frequently than the top 20 real news stories from sites like the *New York Times* and *Washington Post* (Silverman, 2016). Why is it that misinformation and other forms of “problematic information” (Jack, 2017) tend to circulate further and faster than credible and accurate news and information? One oft-repeated explanation is that misinformation is crafted to be novel and shocking (Vosoughi et al., 2018), or to provoke a strong emotional response that drives online sharing (Martel et al., 2020). However, the content itself is only part of the story. What circulates is also informed by how people conceptualize their online audience and how they wish to present themselves in relation to that audience.

Researchers of science and health communication have been diligently working to understand how best to communicate science online, particularly in response to the current health misinformation environment that has characterized COVID-19 communication (Capurro et al., 2021; O’Hair & O’Hair, 2021; Saitz & Schwitzer, 2020). When it comes to science and health information, the research clearly demonstrates that an information deficit model does not work particularly well to address misinformation (Ecker, 2017; Kitta & Goldberg, 2017). Yet, scholars are only beginning to understand how connections or relationships on social media drive sharing (E. M. Kim & Ihm, 2020). To help build out this work on the role of relationships, we bring the concepts of imagined audiences and impression management to bear upon people’s stated motivations for sharing public health infographics about COVID-19 produced by the World Health Organization (WHO; see Appendices A and B). We selected infographics from the WHO because it is a global health institution that has been significant to COVID-19 public health management. We use qualitative elicitation interviews to understand how people’s conception of their online audience, and self-perception in relation to that audience, influences whether they choose to share health-related information with their networks.

This research shows that people’s conceptualization of their online audience shapes their decisions to share health and science information on social media in three key ways, which we discuss in the findings below. This research has implications for health and science communication on social media platforms. Equipped with a better understanding of how people make decisions about what they will or will not share online can help public health researchers, communicators, and educators develop communications strategies that have the best chance of circulating widely and reaching the largest number of people.

This article begins by outlining the theoretical framework of the imagined audience and impression management, which help us understand why people do or do not share certain information, including the health infographics produced by the WHO. Next, we discuss the methods of data collection and analysis employed in this research, followed by a presentation of our findings. Finally, we show how people’s concepts of their online audience and efforts to manage their self-presentation in relation to it impacts health information sharing, and reflect upon the implications of this for combatting the spread of health misinformation online.

## Theoretical Frame: Imagined Audiences and Impression Management

The imagined audience is a concept that has its roots in the work of Irving Goffman (1959) who posited the metaphor of life as a theater with both a “front stage” and a “back stage” that shapes how people behave. When interacting with others, Goffman (1959) argued that people perform a particular “front stage” version of the self that is conscious of the presence of others and the possibility of their scrutiny. This presentation of the self is negotiated against one’s understanding of the audience with whom they communicate, and people adjust their behavior in an effort to manage how others will perceive them.

The imagined audience, then, is a “mental conceptualization of the people with whom we are communicating” (Litt, 2012, p. 331). It is a construct that the communicator intuitively develops and deploys to help them navigate a variety of social contexts and make decisions about what to say and how to act based upon their perception of who the audience is and what their expectations are. In this sense, the imagined audience “acts as a guide for what [behaviour] is appropriate and relevant” (Litt & Hargittai, 2016, p. 1). It is a tool that helps to preserve a particular self-image (Ihm & Kim, 2018), manage others’ impressions (Ranzini & Hoek, 2017), maintain or strengthen interpersonal relationships (C. Kim & Lee, 2016; Waterloo et al., 2018), and establish legitimacy or credibility in various contexts (Bossio & Sacco, 2017; Jordan, 2020).

The imagined audience is never entirely aligned with who the *actual* audience is for acts of communication (Marwick & boyd, 2011). For instance, we might be overheard or observed by those who are not part of our intended audience, producing unintended effects. However, we also cannot *know* with total certainty the lens through which our audience interprets our communication. In this way, our perception of the audience is always partial, inferred, and illusory, to some degree. It is always, in part, a “fiction” (Ong, 1975) conjured to guide communicative choices. In research on the creative and cultural industries, for instance, various scholars have found that writers and other cultural producers conjure an “ideal” audience and write to their imaginary expectations (Nelson, 2021; Peterson, 2003). This ideal is often modeled off the communicator themselves, evoking an audience of other “like-minded” individuals and communicating with their interests, knowledges, and subject positioning in mind. Nelson (2021), for instance, finds that some journalists “look inward to determine what their audience want[s]” (p. 55), using their own inclinations to guide their editorial decisions. Similarly, in an ethnography of television production, Peterson (2003) argues that producers “imagine others like and unlike themselves, (re)constructing their own identities in the process of constructing the imagined audience” (p. 161 cited in Marwick & boyd, 2011). Marwick and boyd (2011) have noted similar dynamics among Twitter users who conceptualize an audience that is a “mirror-image” (p. 120) of themselves.

In conceptualizing the audience, individuals rely upon contextual cues and feedback to inform their understanding of who this group is. In face-to-face communication, the context, people in the room, body language, and facial expressions of others all offer information to the speaker. This feedback is constantly integrated into the speaker’s imagining of the audience, who adjusts, accordingly. In this way, the imagined audience is not a stable construct; it is mutable and in perpetual flux (E. M. Kim & Ihm, 2020).

On social media people have fewer cues to help them construct their imagining of the audience (Livingstone, 2005). Online, the people who actually see a post are largely invisible to the speaker. The mediating role of the communication technology and the way that social media platforms are designed obscures many members of this group from view (Marwick & boyd, 2014; Papacharissi, 2011). Instead, people rely upon feedback from “engagement” such as likes, retweets, and comments, or platform-provided analytics to understand who the audience is.

Online audiences are also large and diverse in a way that extends beyond our ability to cognitively account for all the members of the potential audience (Litt, 2012) and all the possible interpretations and responses that may come from our expressions. Social media platforms aggregate a diversity of relationships from different spheres of life into one space. Friends, co-workers, family, neighbors, acquaintances, and strangers all converge into a mass of possible audience members and the specificity of social context begins to erode. As others have noted, this “context collapse” (boyd, 2008; Davis & Jurgenson, 2014; Marwick & boyd, 2011) makes it very difficult to tailor a communicative act in such a way as to be suitable for the multitude of relationships and communicative contexts into which an expression may enter. Furthermore, when online speech has the capacity to circulate far beyond the poster’s network of interpersonal relations, it becomes difficult to even conceive of an audience of such size, complexity, and multiplicity. Because of this, as Marwick and boyd (2011) explain, people frequently make decisions about what to share based upon a conceptualization that is far narrower in scope. They write that while we may intellectually understand our online audience as “potentially limitless” (Marwick & boyd, 2011, p. 115), we tend to “act as if it were bounded” (p. 115), drawing upon specific referents to make decisions about what to share and the style in which to do so. People tend to invoke the image of certain people and recall particular interactions that become representative of the potential audience and delimit the boundaries of what is appropriate and relevant to say (Cook & Teasley, 2011; Litt & Hargittai, 2016).

As we can see, a person’s vision of *who* their audience is powerfully shapes their decisions about what to share on social media. However, these decisions also crucially depend upon how the speaker hopes to be perceived in relation to the audience. Goffman (1959) notes that all acts of communication are shaped by efforts to manage others’ impressions of the speaker. “Impression management” describes the way that individuals perform the self in public settings, seeking to project a particular self-image and to control how others will perceive them. People work to manage how others will view them through communicative practices of selective self-presentation and strategic disclosures (Kerr et al., 2015). They perform the self, conscious of the presence of others, and attempting to construct a particular image of themselves within the minds of audience members.

In the context of online communication, Papacharissi’s (2002) analysis of personal webpages, for instance, demonstrates how people use text, hyperlinks, images, and colors as “expression equipment” to communicate their interests, status, and personality to their audience. Similar logics are at play with regard to how people manage their social media profiles, where decisions about what to post are inflected by the self-image that the user hopes to establish in the minds of their online audience (Papacharissi, 2011). Such impression management strategies help people to “achieve smoother interpersonal relations or important social goals” (Kerr et al., 2015, p. 471). They are self-making activities that reflect a person’s desire to lay claim to particular identities, establish membership to certain groups, or to acquire status in various contexts and social interactions.

The theoretical scaffolding of imagined audiences and impression management provide a useful frame through which to understand interviewees’ comments about whether they would share the COVID-19 public health infographic from the WHO. We ask the following: how do interviewees imagined audiences and their self-presentation in relation to that audience shape decisions regarding the sharing of WHO public health information?

## Methods

### Data Collection

Between 22 June and 6 July in 2020, we conducted one-on-one online interviews with participants using BlueJeans, a video conferencing application. For the interviews, we used a semi-structured interview protocol that aimed to elicit responses from participants about their engagement with COVID-19 information online. During the interview, participants were presented with two infographics shared by the WHO on Facebook in early 2020 which they were asked questions about: one focused on the community health benefits of vaccines, generally speaking, and the other focused on mask wearing (Appendices A and B). In this article, we focus on their responses to questions about whether they would share the infographics with others online and their reasons to do so or not.

This method of artifact elicitation differentiates this study from other audience research in two noteworthy ways. First, audience research typically operates retroactively, asking the poster about the imagined audience after a particular post has been made (Litt & Hargittai, 2016; Marwick & boyd, 2011). By contrast, we asked participants whether they *would* share the WHO infographic, offering an opportunity to reflect on the hypothetical and contextual factors that would shape their decision to do so or not. Second, audience research also tends to consider the imagined audience in relationship to posts that are made (Semaan et al., 2015), and less frequently do they attend to the question of who the imagined audience is in decisions not to post. In these two ways, this study’s method provides a unique contribution to this field of inquiry.

### Participants

To recruit potential participants, we created and posted a Facebook advertisement inviting people to participate in the study. The invitation targeted Canadians who were at least 18 years of age at the time of data collection who had recently engaged (e.g., reading an article, watching a video, sharing, or commenting on a post, clicking on a link) with COVID-19 information online. Participants were compensated with either a CAD$25 donation to Food Banks Canada or a CAD$25 gift certificate. To ensure that we would conduct interviews with a diverse pool of participants, we used purposeful sampling. Selected participants comprised a group of individuals of different ages, gender, and educational level.

We conducted interviews until we reached saturation, an approach that is typical in qualitative research (Baker & Edwards, 2012). Data saturation is identified by recurrent repetition of data, deeming collection of more data unnecessary. In total, 27 individuals were interviewed. Table 1 presents the list of pseudonymous participants in alphabetical order. Of those who took part in the study, 17 were female, nine were male, and one chose not to disclose. Ten participants were between 18 and 29 years old, seven were between 30 and 45, six were between 46 and 59, and four were 60 or above. More information on participants can be found in Table 1.

**Table 1.** Participants.

Pseudonym	Province	Age	Gender	Highest level of education
Anna	AB	18–29	Female	Master’s degree
Beth	ON	30–45	Female	Associate degree
Bill	BC	30–45	Male	Bachelor’s degree
Cecilia	BC	30–45	Female	Some college
Chantal	ON	30–45	Female	Bachelor’s degree
Dawn	ON	60 or above	Female	Bachelor’s degree
Derek	BC	30–45	Male	Bachelor’s degree
Dorian	ON	18–29	Male	Bachelor’s degree
Eli	ON	60 or above	Male	Bachelor’s degree
Genevieve	MB	18–29	Female	High school or GED
Jess	ON	18–29	NA	Bachelor’s degree
Joan	ON	60 or above	Female	Bachelor’s degree
Linda	AB	30–45	Female	Bachelor’s degree
Megan	MB	46–59	Female	Bachelor’s degree
Olivia	BC	18–29	Female	High school or GED
Roger	ON	46–59	Male	Bachelor’s degree
Rosa	ON	46–59	Female	Doctoral degree
Ruby	BC	18–29	Female	Associate degree
Ryan	ON	18–29	Male	High school or GED
Sandra	ON	46–59	Female	Professional degree
Sarah	ON	18–29	Female	Bachelor’s degree
Sasha	ON	46–59	Female	Some college
Sebastian	ON	18–29	Male	Bachelor’s degree
Shawn	AB	46–59	Male	Some college
Sophia	ON	18–29	Female	High school or GED
Tina	BC	60 or above	Female	Some college
Wallace	BC	30–45	Male	Master’s degree

### Data Analysis

We imported the interview transcripts into NVivo and analyzed them iteratively. In addition to the thematic analysis, we also used descriptive statistics. The iterative analysis was conducted as follows:

1. Two researchers individually coded three interviews. To conduct the open coding, the coders used the constant comparative approach (Glaser & Strauss, 1967), which allows for the identification of emergent themes.
2. Following the initial independent coding session, a group discussion was held. During the discussion, a consensus about the coding scheme was achieved among all the members of the team (McDonald et al., 2019).
3. Next, remaining interviews were shared between the two researchers for coding.
4. Once coding was concluded, the researchers met for another round of evaluation of the coding scheme. To ascertain intercoder reliability, coders exchanged coded data and reviewed codes and themes.

### Rigor and Trustworthiness

To minimize the possible influence of bias on the analysis of the data, we took several steps. First, the coders individually reviewed interview transcripts to get an initial idea of possible patterns in the data. Next, the coders coded three interviews separately and did not maintain contact during that process. Once this initial stage was concluded, the coders met to discuss emerging codes. This stage revealed minimal differences in coding (mainly labeling); these were discussed prior to the coding of the remaining interviews, which commenced once agreement was reached. In addition, once the coding of all interviews was concluded, coders traded interviews to ascertain that the codes were agreed upon by both. Finally, we have employed thick description of the stages of analysis to make it possible for other researchers to evaluate the applicability of our study to their experiences (Merriam, 1995).

## Findings

Interviewees’ willingness to share the WHO infographics was negotiated against who they perceive their audience to be and how they wish to be perceived in relation to that audience. The findings can be grouped into three distinct and prominent themes regarding how interviewees conceptualize the audience and the role that this construct plays in making decisions about sharing the WHO infographics. First, many interviewees conjured an audience of self-similar peers, who were “in the know” about COVID-19; second, those who might share the infographics conjured a specific and contextual audience who “needed” the information; and finally, those who said they would share the infographics most frequently conjured an abstract audience of “the public” or “my community” to explain that decision.

### The Mirror-Image Audience and Non-Sharers

Many interviewees evoked a narrow and homogeneous audience of people who shared the same views and have the same knowledge of the information regarding COVID-19. They tended to describe an audience that was like them, a “mirror image” (Marwick & boyd, 2011) of themselves. For instance, one interviewee explained their decision to not share the information:

I feel it would be really redundant for my people. Because I feel like we’re on the same page. [. . .] I think sharing something like this [is] trying to convince someone on something. You’d want to do it for opinions that you don’t think they have. But [on social media platforms] like Facebook or anything, I guess towards your friends or online friends or acquaintances, generally they have the same opinions as you.

The audience constructed in this case is one that holds the views to that of the poster and has similar knowledge of COVID-19 public health guidelines. Because the poster already knows this information, this self-similar vision of the audience acts as a rationale for not sharing the WHO infographic. Other interviewees provided similar explanations for their decision not to share. One commented,

I also feel that most of my people, my people, people in my group, my people, my posse, we all know that we’re not doing this for ourselves. Like, this isn’t a message that they actually need to have [. . .] I think that they know that.

In this excerpt, the audience is made up of “my people,” demarcating the interviewee and their audience as a collection of COVID-19 savvy citizens who are “in the know” about COVID-19. This vision of a shared subject position and knowledge base is evoked as an explanation for the decision to not share, a fact that is visible in the way that this interviewee includes themself in explaining that “we all know that.” This was a common construct conjured by many interviewees as the rationale for their decision to not share. Another commented, “Most of the people that I relate to probably would agree with this content anyways, so I think it wouldn’t be relevant for the demographics that I associate with on social media.” This sentiment was also present in comments such as “I think my group already got it some time ago”; “most people I know are not against vaccines”; or “I feel like the people who will see my posts hopefully are for vaccination anyway.” The choice to not share the WHO infographic was frequently rationalized with reference to a shared subject position and group identity between the poster and their imagined audience, one that was knowledgeable and already “got it.”

In one instance, an interviewee mentioned specific contacts who they characterize as “conspiracy anti-vaxxers,” yet they still conjured an audience that shares their views and invoked this as their justification for not sharing the WHO infographic. They explained, “I don’t have a lot of—aside from some way out there conspiracy anti-vaxxers—I don’t have a lot of anti vaxxers in my immediate circles.” Interestingly, despite recalling contacts who hold conspiratorial views about vaccines, this interviewee establishes a boundary around “my immediate circles” that creates distance between the poster and this potential audience member as justification for the choice to not share this information.

### The Contextual Audience and Conditional Sharers

Among interviewees who said they might share the WHO infographic, this choice was explained as conditional upon whether or not they could call to mind an image of someone who “needed it.” The demonstrated need for, and therefore relevance to, the imagined audience was an important contextual cue required to prompt sharing. As one interviewee explained,

I would, I guess, if people refuse to wear a mask around me. But I think most people I know, like, at work, and family, [and] friends, like, they know that they have to wear a mask even though it’s very uncomfortable. So I don’t think I would have to send a fact to them because people know to do that.

These interviewees drew upon specific referents such as “friends,” “family” or “co-workers” in building the mental construct of their online audience and drew upon specific interactions to make evaluations about whether or not to share the WHO infographics. As another interviewee commented,

If one of my friends, or one of my coworkers, or somebody in my life comes to me and says, “Oh, wearing a mask for a long time causes this, this and this,” I’d be like, “No, this is actually not the case.” And I’d probably send them this after giving them an explanation. Or if I see it a lot on my newsfeed or if I hear it a lot from people, I would probably share this, yeah.

These interviewees’ comments suggest that they are relying upon specific cues from the audience to demonstrate the need for this information to prompt sharing. The way that evidence of need is required to prompt sharing is also visible in comments such as “Maybe if I just had a conversation with someone. Maybe I would like share it right after or something like that.” Another explained similarly, “If somebody had said like, Oh, I know that’s CO2 is caused by wearing masks, I would send them this.” A third interviewee, who had shared the infographic prior to the interview, explained that they made that decision “because I have seen people express the very concerns that it is addressing.” These comments reflect an imagined audience that is specific, concrete, and local, and suggests that the poster understands their role in relation to this audience as conversational and contextual.

### The Abstract Audience and Unprompted Sharers

Those who reported, unequivocally, that they would share the WHO infographic more frequently invoked a generalized and abstract audience. They envisioned an audience of “the public,” “people around me,” “the community,” “a lot of people,” or “some people” to guide their decisions about sharing. As one interviewee explained,

I wouldn’t hesitate to share something like this because, again, it’s a common sense message. It’s a trusted source. And you know, it is the sort of thing that needs to be reinforced by sharing through the community.

This more abstract imagining of “the community” as the audience for this information is here used to explain this interviewee’s decision to share the WHO infographic. Another interviewee made similar comments, explaining,

Because I think, my like good citizen sense would be [that] even though I’ve never seen anybody on any of my social media feeds make the claim that CO2 intoxication happens by wearing a mask, I would still put it out there [. . .] Yeah, I would share this because it’s good information.

In conjuring the image of the “good citizen,” this interviewee situates herself as part of a broader and more abstract community of citizens in a society. Her decision to share is informed by a broader conceptualization of the potential audience for this message and its impacts beyond her immediate network of interpersonal relations. Another interviewee described their logic for sharing the post in terms of their work in the health care system:

Probably. [. . .] I do think that it’s a fundamental point. And the message that it’s trying to get across is very valuable. Especially from what I’ve seen in health care. A lot of people seem to have a lot of questions when it comes to the efficacy or the effectiveness of masks. And I feel like if this was given to them, they may actually find it more comfortable to actually wear a mask and and not be bothered by any fear of, you know, being poisoned by CO2 from covering their face. So yeah, I would share it.

For this interviewee, the audience for the message extends beyond their personal network to members of the community who they have seen or heard of in the health care system. This broader conceptualization of the audience, once again, seems to be more strongly associated with the decision to share.

## Discussion: Imagined Audiences and Impression Management in How Health Information Spreads on Social Media

As outlined earlier, many interviewees described an audience of self-similar peers for what they post online, and the cohesion and uniformity of this audience was crucial to how they justified their decision to not share the WHO infographics (i.e., “My audience is like me. I know this information, therefore they know this information”). In part, this “mirror-image” audience (Marwick & boyd, 2011) likely testifies to the impact that algorithmically curated social media environments have on how people conceptualize their online audience. Social media platforms deploy user data and algorithmic curation to produce a highly personalized experience for each user. Generally speaking, these environments are designed to deliver users more of what they like. As various scholars have noted, these mechanisms can have the effect of reinforcing established views and beliefs by elevating agreeable perspectives, while limiting exposure to alternatives (Acemoglu et al., 2021; Törnberg, 2018). Our interview data suggest that these mechanisms may be contributing to a sense of uniformity and cohesion between oneself and their online audience that dissuades the sharing of important public health information that the poster does not find novel. In conceptualizing their online audience as a reflection of themselves, these interviewees are unable to envision a scenario where the act of sharing might bring this information into contexts where it could be useful, and equally, uninterested in entertaining the notion that someone in their network might have been affected by COVID-19 misinformation.

Furthermore, there is a clear effort to manage one’s self-image in relation to the online audience of peers in the rationales provided by these non-sharing interviewees. They repeatedly referenced “my people,” “my inner circle,” “my group,” or “the demographics that I associate with” in their explanations, establishing their own group affiliation and belonging to a community of people who are “in the know” about COVID-19. These comments suggest that individuals are considering how their own identity and membership to the group of “knowledgeable people” is established or undermined through their posting decisions. The act of sharing and not sharing here operates as a mechanism for ideal self-presentation in the eyes of the audience. Behind the justification that “we already know that,” there is a sense of anxiety about how they will appear to others if they were to share information that they already know: “Will I appear as if I didn’t know this and mark myself as an outsider to the group who is knowledgeable about COVID-19?” Various studies have found that the risk of damaging one’s self-image and relationships discourages online sharing of political opinions (Hoffman & Lutz, 2017; Liu et al., 2017). When people perceive that their views deviate from that of the majority, the fear of being ostracized produces a “spiral of silence” (Hampton et al., 2014; Noelle-Neuman, 1974) that has various consequences for the quality and diversity of discussion within the public sphere (Matthes, 2015; Stoycheff, 2016). A similar dynamic may be on display in this case study, where the risk of being perceived as unknowledgeable about COVID-19 to one’s peers, and therefore cast out of the group who are “in the know,” undermines the widespread circulation of important public health information. If this is the case, public health communicators should consider how the impression management strategies of social media users may conflict with the simple public health messaging strategies of institutions like WHO, particularly where the self-image people hope to project is to be knowledgeable on the topic.

The fact that many interviewees required evidence that this information was needed by someone they know before they would share it further illustrates that, for many, social media sharing is not a practice of information dissemination, but one of self-performance and relationship building (E. M. Kim & Ihm, 2020), shaped by the context of one’s lived experience and interactions with others. For many interviewees, in the absence of the appropriate experience or interaction that would make this information topical and conversational, the WHO infographics did not make sense to share. This relational approach to sharing demonstrates that good public health information does not necessarily circulate on the basis of its quality, accuracy, or the credibility of the source. It is shared as part of a conversation, often as a response or a corrective to the presence of inaccuracies and misinformation in one’s network of relations. These interviewees’ explanations indicate that they are prepared to engage in debunking practices, seeking to correct falsehoods and misunderstandings as they present themselves, however they are, generally, more resistant to engaging in pre-emptive information sharing practices, such as circulating scientifically sound information *before* witnessing inaccuracies in their social network.

Unfortunately, such sharing behaviors sit at odds with recent research on the efficacy of debunking practices. While debunking is a commonly used tactic to fight misinformation, there is a growing body of research that shows that it is more difficult to correct a falsehood after someone has been exposed (De Keersmaecker & Roets, 2017; Lewandowsky et al., 2012). Once a particular narrative has been adopted it becomes increasingly difficult to dislodge over time (Jolley & Douglas, 2017). In this way, if people only share content from sources like the WHO after seeing evidence of misinformation in their social networks (as many interviewees told us they would), it may be too little too late. The reactive approach to health information sharing that interviewees articulated is less effective than sharing *before* a contact has been exposed to misinformation.

Finally, the interviewees who did report that they would share this information more frequently evoked an abstract audience of “the public” or “the community” than did those who would not share this information, and perceived themselves to be acting in the role of “good citizen” in relation to their audience. This more abstract imagining of the online audience may function as a mechanism that helps participants overcome the risk of damage to their self-image through posting. This finding suggests that a more abstract imaginings of the online audience are more conducive to the sharing of health information. However, this conceptualization of the audience as “the public” may be related to the specific platform that interviewees were envisioning in making decisions about sharing. The features and cultural norms of different platforms inform how users conceptualize their audience with consequences for their sharing behaviors (Choi & Lee, 2017; Ihm & Kim, 2018; Litt, 2012). For instance, on asymmetrical platforms such as Twitter or Instagram users can choose to follow others without consent or reciprocity, while on symmetrical platforms like Facebook or WeChat, contacts must be accepted into a social network. On asymmetrical platforms, then, users may be more inclined to view their audience in broader and more abstract terms with different sharing practices to those on symmetrical platforms who may conceptualize the audience in more interpersonal terms (Choi & Lee, 2017).

In practice, this has implications for how organizations design their messages for circulation across social media environments. Organizations like the WHO that wish to encourage the spread of accurate health information online should make sure that they are tailoring their approach to specific platforms, keeping in mind the way that different environments have different functionalities that structure how people conceptualize and interact with their audiences. In this instance, although we originally sourced the WHO infographics from Facebook, the simple public health messaging of this content may have been more suitable to and widely shared on Twitter, where the technological and imagined affordances (Nagy & Neff, 2015) of the platform lend themselves to a more generalized audience imagining.

One of the well-established and commonly deployed strategies to contend with misinformation is to create and disseminate accurate information on social media, such as the WHO COVID-19 infographics (see also https://www.scienceupfirst.com/). However, if regular social media users do not also share this information, it limits its circulation and the possibility that it will reach the individuals and communities who need it. Our data reflect that people’s social media sharing decisions are negotiated against how they conceptualize their audience and themselves in relation to it. These findings have important implications for efforts to combat the impact of health misinformation. First, they underscore the need to consider what public health messaging can contribute to the self-image of social media users to facilitate widespread sharing. For instance, public health communicators might consider how messaging could make use of novelty, specificity, and intricacy in addition to strategies that foreground the principles of simplicity, clarity, and universality. Second, the fact that a portion of interviewees reported that they would share these infographics in response to misinformation indicates a need for more public education regarding the inefficacy of debunking practices after false information has been accepted, and the importance of being pre-emptive to inoculate against misinformation.

## Conclusion

In this research, we examined how social media users imagined audiences, and their self-presentation strategies in relation to that audience, shape people’s health information sharing practices online and considered the implications of this for efforts to combat the spread of health misinformation on social media. Using infographics produced by the WHO and shared on Facebook, we conducted elicitation interviews to determine whether people would share this content with others on social media, and why or why not. Responses revealed that whether a person would share WHO infographics depends on their view of their own imagined social media audience and their self-perception in relation to that audience. The bulk of our interview participants constructed an imagined audience of self-similar peers, and reported that they would not share the WHO infographics because they are part of a group that is “in the know” about COVID-19. Conditional sharers, who reported that they might share the WHO infographics, imagined a concrete audience and sharing practices that were relational, local, and conversational. For unprompted sharers, who reported that they would share the WHO infographics, they conceptualized their audience as a broader community of the public, and themselves as “community members,” “citizens,” or “educators” for sharing and contributing to the circulation of this information. These findings have important implications for efforts to combat health misinformation on online platforms through the circulation of scientifically sound public health information. First, they underscore the need to consider what public health messaging can contribute to the self-image of social media users. Second, it underscores that misinformation researchers need to do more to educate the public about the inefficacy of debunking and the importance of being pre-emptive in spreading credible information. Finally, our findings also show that communicators who wish to counter health misinformation with content that others are likely to share online need to consider not only the clarity of the content, but also try to appeal to those people and platforms that are more likely to serve a broadcast function within a larger community.

## Limitations and Future Research

These interviews were conducted at the beginning of the pandemic in 2020. It is possible that the presumption that one’s social media audience is a reflection of oneself has been troubled. There is much anecdotal evidence to suggest that many people are having the experience of seeing that there is more diversity in their network than they might have initially realized. Future research should examine changes to sharing behavior over the course of the pandemic as the situation evolved. Furthermore, the participants who self-selected into our study group were also fairly uniform with respect to culture, whiteness and education level, and thus reflect the needs and experiences of only one group. It remains to be seen whether these emerging trends play out over a larger scale and with different diverse groups, particularly in locations where the WHO may have an outsized importance on the ground rather than just in the media. As such, future research, using a survey or other larger scale methodology, should look to tease out any differences in how diverse cultural groups and nationalities imagine their audience and their own role in sharing health information relative to that audience.

Unfortunately, this study did not ask participants to specify about the particular platforms they use, or which they were envisioning when discussing their intentions to share or not share. The researchers opted, instead, to ask a broad question about sharing on “social media” and leave it to the interviewee to interpret. This has made it difficult to surmise the role of different platform affordances in shaping sharing practices for health information. Future research should account for platform specificity in the research design.

Finally, the fact that a broader mental construct of the audience was more consistently aligned with an intention to share than more bounded visions of the audience, raises additional questions to future research. For example, does a broader mental construct of the imagined audience lend itself more readily to proactive sharing of “good” scientific information? If so, how best to cultivate this imagined audience in the messaging from organizations like the WHO?
